# Chronic vulvar pain in a cohort of post-menopausal women: Atrophy or Vulvodynia?

**DOI:** 10.1186/s40695-016-0017-z

**Published:** 2016-06-09

**Authors:** Susanna D. Mitro, Siobán D. Harlow, John F. Randolph, Barbara D. Reed

**Affiliations:** 1 Department of Epidemiology, School of Public Health, 1415 Washington Heights, Ann Arbor, MI 48109 USA; 2 School of Medicine, University of Michigan Ann Arbor, Ann Arbor, MI USA

**Keywords:** Female Sexual Function Index, Vaginal Dryness, Multinomial Logistic Regression Model, Serum Hormone Level, Vulvar Pain

## Abstract

**Background:**

Although postmenopausal vulvar pain is frequently attributed to vaginal atrophy, such symptoms may be due to vulvodynia, a chronic vulvar pain condition. Given the limited research on vulvodynia in postmenopausal women, the objective of this study was to provide preliminary population-based data on the associations of vaginal symptoms, serum hormone levels and hormone use with chronic vulvar pain in a multiethnic sample of post-menopausal women.

**Methods:**

We used data from 371 participants at the Michigan site of the Study of Women’s Health Across the Nation (SWAN) who participated in the 13th follow-up visit. Women completed a validated screening instrument for vulvodynia and provided information on additional vaginal symptoms as well as demographic characteristics, and hormone use by questionnaire. Blood samples were obtained to assess hormone levels. We compared women who screened positive for vulvodynia and women with past or short-duration vulvar pain to women without vulvar pain, using Chi-squared and Fisher’s Exact tests. Relative odds ratios and 95 % confidence intervals were calculated using multinomial logistic regression models adjusting for age, body mass index, and race/ethnicity.

**Results:**

Current chronic vulvar pain consistent with vulvodynia was reported by 4.0 % of women, while 13.7 % reported past but not current chronic vulvar pain or short-duration vulvar pain symptoms. One quarter of women who reported current chronic vulvar pain did not report vaginal dryness. Women with current chronic and with past/short duration vulvar pain symptoms were more likely to have used hormones during the preceding year than women without vulvar pain symptoms (13.3 %, 17.6 %, 2.0 %, respectively; *p* < .01). Increased relative odds of current vulvar pain symptoms were associated with each log unit decrease in serum dehydroepiandrosterone-sulfate, estradiol and testosterone levels at the previous year’s visit.

**Conclusion:**

Some women who experience chronic vulvar pain symptoms do not report vaginal dryness, and others report continued or first onset of pain while using hormones. Vulvodynia should be considered in the differential diagnosis of postmenopausal women presenting with vulvar pain symptoms.

## Background

Although vulvar pain symptoms can occur at any time over the life span, it is not uncommon for symptoms to begin for the first time after menopause [1–3]. In fact, the prevalence of chronic vulvar pain in mid-life women has been estimated to be 8.9-38 % percent, making chronic vulvar pain a major health concern for women in this age group [4, 5]. However, despite recognition of the burden of chronic vulvar pain symptoms in the midlife, research on vulvodynia, a chronic pain condition characterized by pain in the vulva, has mainly focused on premenopausal women [1, 2].

Until recently, research on postmenopausal vulvar pain symptoms has largely focused on vaginal dryness and vulvar atrophy, secondary to estrogen deprivation. However, evidence indicates that postmenopausal vulvar pain may occur for other reasons as well [6–9]. Additionally, women with atrophy do not all experience pain [7], episodes of postmenopausal vulvar pain are not all successfully treated using estrogen therapy [8, 9], and in a recent study serum estradiol, estrone, and progesterone levels of postmenopausal women were not tightly correlated with vulvar pain [3]. These findings suggest that some vulvar pain reported by postmenopausal women may be a condition other than atrophy, such as vulvodynia, and present independent of estrogen-or atrophy-related changes.

This paper evaluates chronic vulvar pain reported by African American and white women participating in the Michigan site of the longitudinal, multiethnic Study of Women’s Health Across the Nation (SWAN). Although the sample size is limited, prospective measurements of age at final menstrual period, symptoms of vaginal dryness, and hormone therapy (HT) use, as well as serum hormone levels obtained prior to the assessment of current pain status, provide preliminary data on the estimated prevalence of chronic vulvar pain, consistent with vulvodynia in post-menopausal women and its association with hormones, HT use and vaginal dryness in this population-based sample of postmenopausal women.

## Methods

This study used data from the Michigan site of SWAN, a multiethnic prospective cohort study addressing health-related changes in the midlife and menopausal transition. The cohort has been described in detail previously [10]. Briefly, in 1996, each SWAN clinic site enrolled white women and one targeted minority population. The Michigan SWAN population, established using a community census, was composed of women aged 42–52 years at baseline, who were not using exogenous hormones at the time of enrollment, had an intact uterus and at least one ovary and had had a menstrual period in the three months before enrollment, were not pregnant or lactating, and self-identified as either white or African American. At baseline and each follow-up visit a blood sample was collected, height and weight measures were taken while demographic characteristics, medication use, and symptoms of vaginal dryness were ascertained by questionnaire. Over the next 17 years women participated in follow-up visits approximately annually. At the 13th follow-up visit in 2012, the Michigan site added several screening instruments for chronic pain conditions including a validated screening instrument for vulvodynia [11].

At baseline, the Michigan SWAN cohort was composed of 543 women, 60 % of whom were African American by design. In 2012, 32 (5.9 %) women had died and 411 (80.4 % of the non-deceased cohort) were still active, 380 (92.5 %) of whom participated in follow-up Visit 13. Nine women who did not answer any questions pertaining to vulvar pain were excluded, leaving 371 wome*N* (61.7 % African American) eligible for this analysis. For analyses including endogenous serum hormone levels, we evaluated hormone levels at Visit 12, to ensure hormone levels preceded the report of vulvar pain status at Visit 13. These analyses include 319 women as we excluded the 37 women who did not have blood drawn and the 15 women who reported HT use at Visit 12.

### Ethics and consent

This study was approved by Health Sciences and Behavioral Sciences Institutional Review Board of the University of Michiga*N* (HUM00083308). Women provided informed consent at baseline and each follow-up interview.

In Visit 13, Michigan participants completed a validated screening questionnaire for vulvodynia [11] that obtained information on symptoms of vulvar pain or discomfort, including date of pain onset, duration of pain, and whether pain continues. We interpret a positive screen in this postmenopausal population to be consistent with vulvodynia but acknowledge that this screening tool may not adequately differentiate vulvodynia from atrophy in this postmenopausal cohort. Therefore we use the term “chronic vulvar pain” in lieu of vulvodynia when presenting the results.

Based on responses to the vulvodynia questionnaire, each participant was categorized into one of three groups: women with current chronic (lasting 3 months or longer) vulvar pain, women who reported ever having chronic vulvar pain in the past or reported having short-duration (less than 3 months duration) vulvar pain symptoms, and women reporting no current or past vulvar pain symptoms. Current chronic vulvar pain was defined by a history of vulvar pain or discomfort at the opening to the vagina that had lasted for at least three months and had been experienced in the preceding three months. The past chronic vulvar pain/short-duration vulvar pain symptom group included women who had a history of vulvar pain lasting for at least three months but who had not experienced pain in the preceding three months and women with current vulvar pain lasting for less than three months. This group represents a heterogeneous symptomatic group who, based on prior work [12], are more likely than the non-symptomatic group to develop vulvodynia, and hence we categorize them separately from the no pain group.

Age was modeled as a continuous variable. Race/ethnicity was self-reported as either white or African American. Measured height and weight were used to calculate body mass index (BMI) (weight in kilograms (kg) divided by height in meters (m) squared). BMI was further categorized as normal weight, overweight, or obese (<25, 25- < 30, and > = 30 kg/m^2^). Socioeconomic status was assessed by self-reported difficulty paying for basics (very hard versus somewhat or not hard) and education at baseline (high school or less versus at least some college). Marital status was categorized as either married or not married.

In addition to questions about vulvar pain, we asked about other specific vulvovaginal symptoms at Visit 13 including self-reported number of days in the past 2 weeks of vaginal dryness, soreness, and irritation categorized into three duration levels (0 days, 1–5 days, or >6 days). In addition, we created variables to reflect whether women ever reported vaginal dryness before, and after, the final menstrual period (FMP) or hysterectomy (yes/no) based on responses at each follow-up visit. Although women were not eligible to enroll in SWAN ff they were using HT, women who began using HT after enrollment remained in the study. Two HT variables were considered: current HT use (yes/no), and ever used HT during the study (yes/no).

At each visit, a fasting blood sample was collected, refrigerated for 1–2 h after collection, and then centrifuged. Serum hormone levels of estradiol (E2), dehydroepiandrosterone-sulfate (DHEA-S), follicle stimulating hormone (FSH), sex hormone-binding globuli*N* (SHBG), and testosterone (T) were determined.

All assays were performed on the ACS-180 automated analyzer (Bayer Diagnostics Corporation, Tarrytown, NY) at the CLASS laboratory at the University of Michigan, utilizing a double-antibody chemiluminescent immunoassay with a solid phase anti-IgG immunoglobulin conjugated to paramagnetic particles, anti-ligand antibody, and competitive ligand labeled with dimethylacridinium ester (DMAE). The FSH assay is a modification of a manual assay kit (Bayer Diagnostics) utilizing two monoclonal antibodies directed to different regions on the beta subunit, with a lower limit of detection (LLD) of 1.05 mIU/mL. Inter-and intra-assay coefficients of variation were 12.0 % and 6.0 %, respectively. The E2 assay modifies the rabbit anti-E2-6 ACS-180 immunoassay to increase sensitivity, with a LLD of 1.0 pg/mL and inter- and intra-assay coefficients of variation averaging 10.6 % and 6.4 %, respectively. The T assay modifies the rabbit polyclonal anti-T ACS-180 immunoassay, with a LLD of 2.19 ng/dL and inter-and intra-assay coefficients of variation of 10.5 % and 8.5 %, respectively. The DHEA-S and SHBG assays were developed using rabbit anti-DHEA-S and anti-SHBG antibodies, with LLDs of 1.52 mcg/dL and 1.95 nM, respectively. For DHEA-S, the inter- and intra-assay coefficient of variation were 11.3 % and 8.0 %, respectively. For SHBG, the inter- and intra-assay coefficient of variation were 9.9 % and 6.1 %, respectively. Duplicate E2 assays were conducted, with results reported as the arithmetic mean for each subject, with a CV of 3-12 %. All other assays were single determinations. Hormone levels below the lower limit of detection were assigned a random number between 0 and the lower limit of detection.

The prevalence of vulvar symptoms overall and stratified by demographic characteristics were calculated and compared using Chi-squared and Fisher’s Exact tests as appropriate. Hormone levels were log-transformed for regression analyses. The median values of the log-transformed E2, DHEA-S, SHBG, FSH, and T at Visit 12 were compared overall and across symptoms groups using Kruskal-Wallis tests. Relative odds ratios (OR) and 95 % confidence intervals (CI) comparing the current chronic vulvar pain and past/short-duration vulvar pain groups to the no vulvar pain group were calculated using multinomial logistic regression models appropriate for outcomes with more than two categories [13]. These models compare odds for reporting current chronic vulvar pain symptoms in relation to the no pain category and odds for reporting past/short-term vulvar pain symptoms in relation to the no pain category. In addition to an unadjusted model, models adjusted for race, BMI, and age were also assessed. Analyses were performed using SAS 9.3 (Cary, NC).

## Results

At follow-up Visit 13, participants ranged in age from 56 to 68 years (median 61.3 years). Of the 371 women eligible for this analysis, 15 women (4.0 %; 95 % CI: 2.5 %, 6.6 %) reported current chronic vulvar pain, 51 (13.7 %; 95 % CI: 10.6 %, 17.6 %) reported past chronic or short-duration vulvar pain, and 305 (82.2 %; 95 % CI: 78.0 %, 85.8 %) reported no vulvar pain. Of the 15 women reporting current chronic vulvar pain, one did not provide an age of symptom onset, four experienced symptom onset before age 45, two experienced onset between age 46 and 55, and the remaining 8 experienced onset between ages 56 and 64 years. Five of the 15 women reported first onset since their previous follow-up visit, representing an incidence of 1.3 %.

Median age, marital status, and proportion sexually active in the previous 6 months did not differ by chronic vulvar pain status (Table 1). However, women with current chronic vulvar pain were more likely to be white, less likely to be obese, and more likely to have completed at least some college compared to women in the other vulvar pain groups (Table 1).

**Table 1:** Demographic and clinical characteristics of women in the MI SWAN population, by self-reported vulvar pain

	Total	Current Chronic Vulvar Pain	Past/Short-term Vulvar Pain	No Vulvar Pain	
Variable	*N* (%)	*n* (%)	*n* (%)	*n* (%)	*p*-*value* ^6^
Race					< .01
White	142 (38.3 %)	10 (66.7 %)	27 (52.9 %)	105 (34.4 %)	
African American	229 (61.7 %)	5 (33.3 %)	24 (47.1 %)	200 (65.5 %)	
Education at Baseline^1 ^					.05
High School or less	108 (30.1 %)	2 (15.4 %)	9 (18.0 %)	97 (32.8 %)	
At least some college	251 (69.9 %)	11 (84.6 %)	41 (82.0 %)	199 (67.2 %)	
Marital Status^2^					.65
Married	188 (55.8 %)	10 (66.7 %)	27 (57.5 %)	151 (54.9 %)	
Not Married	149 (44.2 %)	5 (33.3 %)	20 (42.5 %)	124 (45.1 %)	
Sexually Active in Last 6 Months ^3^					.40
Yes	120 (39.7 %)	7 (58.3 %)	18 (40.0 %)	95 (38.8 %)	
No	182 (60.3 %)	5 (41.7 %)	27 (60.0 %)	150 (61.2 %)	
BMI^4^					.02
<25 kg/m^2^	47 (14.2 %)	3 (21.4 %)	12 (26.7 %)	32 (11.7 %)	
25 - <30 kg/m^2^	81 (24.4 %)	6 (42.9 %)	9 (20.0 %)	66 (24.2 %)	
> = 30 kg/m^2^	204 (61.4 %)	5 (35.7 %)	24 (53.3 %)	175 (64.1 %)	
Currently Use HT					< .01
Yes	17 (4.6 %)	2 (13.3 %)	9 (17.6 %)	6 (2.0 %)	
No	354 (95.4 %)	13 (86.7 %)	42 (82.4 %)	299 (98.0 %)	
Ever Used HT					< .01
Yes	137 (36.9 %)	7 (46.7 %)	30 (58.8 %)	100 (32.8 %)	
No	234 (63.1 %)	8 (53.3 %)	21 (41.2 %)	205 (67.2 %)	
History of Hysterectomy					.21
Yes	63 (17.0 %)	2 (13.3 %)	13 (25.5 %)	48 (15.7 %)	
No	308 (83.0 %)	13 (86.7 %)	38 (74.5 %)	257 (84.3 %)	
*Urogenital Symptoms in previous 2 weeks*					
Dryness					< .01
0 days	272 (73.3 %)	4 (26.7 %)	33 (64.7 %)	235 (77.1 %)	
1-5 days	53 (14.3 %)	3 (20.0 %)	9 (17.6 %)	41 (13.4 %)	
6-14 days	46 (12.4 %)	8 (53.3 %)	9 (17.6 %)	29 (9.5 %)	
Soreness^2^					< .01
0 days	318 (94.4 %)	10 (66.7 %)	44 (93.6 %)	264 (96.0 %)	
1-5 days	15 (4.4 %)	3 (20.0 %)	3 (6.4 %)	9 (3.3 %)	
6-14 days	4 (1.2 %)	2 (13.3 %)	0 (0.0 %)	2 (0.7 %)	
Irritation^2^					< .01
0 days	280 (83.1 %)	8 (53.3 %)	35 (74.5 %)	237 (86.2 %)	
1-5 days	43 (12.8 %)	3 (20.0 %)	10 (21.3 %)	30 (10.9 %)	
6-14 days	14 (4.2 %)	4 (26.7 %)	2 (4.3 %)	8 (2.9 %)	
*History of Reported Dryness*					
Dry Before FMP/Hysterectomy^5^					.46
Yes	126 (46.7 %)	7 (63.6 %)	16 (50.0 %)	103 (45.4 %)	
No	144 (53.3 %)	4 (36.4 %)	16 (50.0 %)	124 (54.6 %)	
Dry After FMP/Hysterectomy^5^					.01
Yes	232 (62.5 %)	10 (90.9 %)	23 (71.9 %)	124 (54.6 %)	
No	139 (37.5 %)	1 (9.1 %)	9 (28.1 %)	103 (34.3 %)	

Missing observations: ^1^12, ^2 ^34, ^3^69, ^4^39 , ^5^101.

^6^All p-values calculated using Chi-squared tests except BMI, Difficulty Paying for Basics, Currently Use HT, Vaginal Dryness, Vaginal Soreness, and Vaginal Irritation, which were calculated using Fisher’s Exact test

Although few women reported current HT use, both the current chronic and past/short-duration vulvar pain groups were more likely than women with no vulvar pain symptoms to report having used HT during the preceding year (Table 1). Women with past/short-duration vulvar pain were more likely to have ever used HT and more likely to have had a hysterectomy than women in the other two groups (Table 1). Two of the 15 women with current symptoms (13.3 %) began HT after pain onset but did not report remission while 3 of the 32 women with current or past chronic vulvar pain symptoms (9.4 %) reported first onset of vulvar pain while taking HT.

Prevalences of self-reported dryness, soreness, and/or irritation were lowest in the no vulvar pain symptoms group and highest in the current chronic vulvar pain group (Table 1). However, although over half of women with current vulvar pain indicated they had “dryness” for over 6 days in the past 2 weeks, approximately a quarter (26.7 %) of women with current vulvar pain did not report vaginal dryness. Similarly, although reporting of “soreness” or “irritation” was most frequent in women with current chronic vulvar pain, over half of the women with current chronic vulvar pain did not report soreness or irritation.

In the multinomial logistic regression models adjusted for race, odds of having current chronic vulvar pain symptoms were significantly elevated in white women, current HT users, and individuals reporting dryness, soreness, or irritation for 6 or more days in the preceding 2 weeks (Table 2). Odds of past/short-duration vulvar pain symptoms were significantly elevated in white women, current and past HT users, women who had had a hysterectomy, and individuals reporting vaginal irritation 1–5 days in the previous 2 weeks. Adjusting the logistic models for age or BMI did not substantially alter results (data not shown).

**Table 2:** Relative odds ratios (OR) and 95 % confidence intervals (CI) for selected variables, adjusted for race

	Current Chronic Vulvar Pain vs none		Past/Short-term Vulvar Pain vs none	
Variable	OR (95 % CI)	*P*	OR (95 % CI)	*P*
Categorical BMI				
<25 kg/m^2^	REF	--	REF	--
25-<30 kg/m^2^	0.96 (0.22, 4.13)	0.34	0.36 (0.14, 0.95)	.21
> = 30 kg/m^2^	0.30 (0.07, 1.35)	0.12	0.36 (0.16, 0.81)	.01
Currently Use HT				
Yes	7.12 (1.27, 39.94)	0.03	10.24 (3.42, 30.68)	< .01
No	REF	--	REF	--
Ever Use HT				
Yes	1.79 (0.63, 5.13)	0.28	2.93 (1.59, 5.40)	< .01
No	REF	--	REF	--
Sexually Active in Last 6 months.				
Yes	2.17 (0.66, 7.11)	0.20	1.04 (0.54, 2.01)	.90
No	REF	--	REF	--
Hysterectomy				
Yes	0.91 (0.20, 4.23)	0.91	1.95 (0.96, 3.97)	.07
No	REF	--	REF	--
Vaginal Dryness				
0 days	REF	--	REF	--
1-5 days	5.47 (1.14, 26.12)	0.03	1.79 (0.78, 4.07)	.17
6-14 days	16.34 (4.53, 58.85)	<0.01	2.22 (0.96, 5.16)	.06
Vaginal Soreness				
0 days	REF	--	REF	--
1-5 days	14.43 (2.95, 70.56)	<0.01	2.46 (0.63, 9.68)	.20
6-14 days	28.28 (2.99, 267.5)	<0.01	(Insufficient data)	.99
Vaginal Irritation				
0 days	REF	--	REF	--
1-5 days	3.30 (0.81, 13.42)	0.09	2.40 (1.07, 5.41)	< .01
6-14 days	12.60 (3.00, 52.86)	0.03	1.53 (0.31, 7.65)	.60

At Visit 12, median serum hormone levels of E2 and DHEA-S tended to be lower (*p* = 0.06) in women who subsequently reported current chronic vulvar pain symptoms at Visit 13 compared to those who reported no vulvar symptoms (Table 3). The unadjusted relative odds of current chronic vulvar pain symptoms versus no vulvar symptoms at Visit 13 were elevated with each log unit decrease in Visit 12 E2, DHEA-S, and T levels (Table 4). After adjustment for age, race and BMI, the odds remained elevated only for DHEA-S and T. FSH and SHBG levels were not associated with chronic vulvar pain. In an exploratory analysis we evaluated longitudinal endocrine patterns prior to pain onset in the five women reporting new onset chronic pain symptoms at visit 13. From Visit 10 to Visit 12, three of the five experienced a sharp drop in E2 levels (defined < =15 % of the Visit 10 level at Visit 12) as did only 12 of 256 women without symptoms.

**Table 3:** Serum hormone levels (median and interquartile range (IQR)) at Visit 12 for all women not using hormonal therapy, by self-reported vulvar pain symptoms at Visit 13

	Total	Current Chronic Vulvar Pain	Past/Short-term Vulvar Pain	No Vulvar Pain	
Variable	Median (IQR)	Median (IQR)	Median (IQR)	Median (IQR)	*p*-*value*
Age (yr)	61.3 (59.4-63.7)	59.3 (58.6-60.4)	61.4 (60.0-63.7)	61.3 (59.4-63.7)	.23
*Hormones* (*log transformed*)					
	*N* = *319*	*n* = *12*	*n* = *38*	*n* = *264*	
E2 (average, pg/mL)	19.8 (12.0-27.2)	15.2 (3.7-24.4)	23.2 (18.8-26.9)	19.4 (12.0-27.3)	0.06
DHEA-S (ug/dL)	63.7 (36.8-88.6)	28.5 (8.0-73.1)	62.0 (49.3-83.2)	65.0 (37.7-90.1)	0.06
FSH (mIU/mL)	52.2 (36.8-70.7)	55.4 (38.7-64.6)	52.7 (31.5-79.6)	51.5 (36.9-70.6)	0.92
SHBG (nM)	48.8 (35.7-68.7)	56.4 (31.8-94.1)	53.1 (34.8-68.5)	48.8 (35.8-67.8)	0.83
T (ng/dL)	49.8 (38.5-61.8)	45.2 (22.5-54.9)	54.7 (41.7-65.4)	49.8 (38.7-61.6)	0.22

**Table 4:** Relative odds ratios (OR) and 95 % confidence intervals (CI) for having chronic vulvar pain by log-transformed serum hormone levels at Visit 12 among women not using hormones

Hormone (log)	Unadjusted Model		Adjusted Model*	
	Current Chronic Vulvar Pain vs none	Past/Short-term Chronic Pain vs none	Current Chronic Vulvar Pain vs none	Past/Short-term Vulvar Pain vs none
	OR (95 % CI)	OR (95 % CI)	OR (95 % CI)	OR (95 % CI)
*Visit 12* (*N* = *319*)				
E2 (average, pg/mL)	0.49 (0.28, 0.86)^**^	1.37 (0.86, 2.19)	0.58 (0.32, 1.04)	1.52 (0.92, 2.50)
DHEA-S (ug/dL)	0.46 (0.29, 0.75) ^***^	1.09 (0.69, 1.73)	0.45 (0.28, 0.72)^***^	1.07 (0.66, 1.74)
FSH (mIU/mL)	1.13 (0.39, 3.28)	1.11 (0.60, 2.05)	0.76 (0.28, 2.10)	1.15 (0.56, 2.39)
SHBG (nM)	1.45 (0.49, 4.31)	0.99 (0.52, 1.89)	1.00 (0.29, 3.47)	0.99 (0.47, 2.11)
T (ng/dL)	0.13 (0.04, 0.48) ^***^	1.38 (0.55, 3.47)	0.14 (0.04, 0.56)^***^	1.39 (0.53, 3.68)

* adjusted for age, categorical BMI, and race

^**^ *p* = .01; ^***^ *p* < .01

## Discussion

Postmenopausal women are as likely as younger women to report chronic vulvar pain consistent with vulvodynia [4, 14, 15]. This study is one of the first population-based studies to examine the association between symptoms of vaginal dryness, serum hormone levels, hormone use and chronic vulvar pain symptoms in postmenopausal women. We found that women with current chronic vulvar pain symptoms often experienced pain onset prior to menopause. Women with current chronic pain were more likely than women without such pain symptoms to be using HT, and some reported the onset of vulvar pain symptoms while already taking HT. Despite the possibility of inadequate hormonal treatment in some cases, vulvar pain unresponsive to HT further supports the presence of a chronic pain condition such as vulvodynia that is likely to require alternative, non-hormonal, treatment modalities. Notably, more than a quarter of women who reported current chronic vulvar pain did not report vaginal dryness, a common complaint associated with vaginal atrophy. Lower average DHEA-S, and T levels prior to ascertainment of vulvar pain symptoms were associated with elevated odds of subsequently reporting chronic vulvar pain, further supporting that for some, hormonal levels may contribute to symptoms experienced. These results provide additional evidence that chronic vulvar pain in postmenopausal women has a heterogeneous etiology and, in many women, may not be explained by estrogen deficiency-related atrophy alone [6, 14, 15].

In this sample of postmenopausal women, the prevalence and incidence of chronic vulvar pain was somewhat lower than that observed in other studies. One previous study reported prevalences in women age 40–65 years of 13.9 % and 8.9 % among women who had and had not used HT, respectively [4, 5], and an incidence of approximately 3.3 per 100 person-years in women age 50 and older [16]. A second paper reported a prevalence of vulvovaginal symptoms suggestive of atrophy in 38 % of women aged 45–65 [5]. The low prevalence of chronic vulvar pain reported in this study reflects the large proportion of African Americans who have been shown to be less likely than white women to report chronic vulvar pain. Vulvodynia is more prevalent in white women than African American women in most [2, 4, 16], although not all studies [17]. The low prevalence reported here also reflects the older average age of the study population, as previous reports have indicated that vulvodynia prevalence and incidence decline with age, especially if those not having sexual intercourse are included in the analysis [4, 16].

Previous research has suggested that a drop in estrogen may be associated with onset of chronic vulvar pain that will not necessarily be reversed by subsequent estrogen supplementation [8]. We observed that lower levels of E2, DHEA-S, and T were associated with increased odds of reporting current chronic vulvar pain, although only DHEA-S and T remained significant after adjustment. When we evaluated longitudinal endocrine patterns prior to pain onset only in the five women reporting new onset chronic pain symptoms at visit 13, three of the five had experienced a prior sharp drop in E2 levels compared to just five percent of women with no symptoms. Although consistent with the theory that a variable hormonal environment may contribute to chronic vulvar pain, we observed only a small number of new onset cases. Further study of the relationship between longitudinal endocrine patterns and risk for chronic vulvar pain is warranted.

Lower DHEA-S levels at visit 12 were associated with higher odds of reporting current chronic vulvar pain symptoms, a finding that should be explored in future studies. An association between low DHEAS and sexual dysfunctio*N* (as measured by the Female Sexual Function Index) [18] and a weak association of serum androgens and sexual well-being in women with premature ovarian failure have been reported [19]. However, a mechanism to explain a direct relationship between DHEAS and vulvar symptoms is unclear. The topical application of DHEA to the vagina in women with severe atrophy has been reported to improve all domains of sexual function, including pain with sexual activity, in controlled clinical trials [20–22], potentially due to local conversion to androgens and estrogens. Future studies are needed to confirm and further assess these findings.

This study adds to the literature indicating that postmenopausal vulvar pain may be caused by factors other than vulvovaginal atrophy [6–9, 14, 15]. Hormone use did not always prevent symptom onset and was not associated with symptom remission in all women. However, as the vulvodynia screening instrument was administered at only Visit 13, timing of vulvar pain onset was ascertained by retrospective report. Also, information on details of HT such as dose, route of administration, indication and duration of use was limited or unavailable; thus, we are not able to assess adequacy of treatment for presumed estrogen deficiency. However, those with vulvar pain symptoms secondary to atrophy who had been adequately treated with estrogen would not be included in the chronic vulvar pain group–hence only those with persistent symptoms despite hormone therapy, and those with persistent symptoms who have not taken HT, were included in the chronic vulvar pain group.

This analysis was constrained by the limited sample size, particularly the small number of women with current vulvar pain symptoms meeting our screening criteria. As a categorization of chronic pain requires a minimum duration of three months and categorization as a past case depends on participant recall, it is possible that some participants forgot to report past episodes, thus attenuating the findings. Nonetheless, a unique strength of this study is the availability of longitudinal data on HT use, serum hormone levels, and self-reports of vaginal dryness in postmenopausal women within a defined timeframe after the final menstrual period permitting a more detailed, though preliminary, look at the relationship between vaginal symptoms, hormone levels and vulvar pain in a population-based, multi-ethnic sample of midlife women. Future studies might consider evaluation of additional pain symptoms such as dyspareunia in relation to reporting of chronic vulvar pain.

## Conclusion

This preliminary but rich longitudinal population-based study adds to the growing literature suggesting that vulvar atrophy may not be the sole cause of postmenopausal vulvar pain. Postmenopausal women may be experiencing new onset, exacerbated and/or long-term chronic vulvar pain consistent with a diagnosis of vulvodynia. Health care providers should consider and evaluate for vulvodynia when treating postmenopausal women with chronic vulvar pain, especially those women who fail to respond to HT. The best tool for distinguishing if chronic vulvar pain consistent with both atrophy and vulvodynia will respond to HT is to give a trial of HT, followed by alternative vulvodynia treatments in those not responding. Future research should focus on the diagnosis and treatment of women who do not respond to this intervention.

### Abbreviations

BMI, body mass index; DHEA-S, dehydroepiandrosterone-sulfate; E2, estradiol; FSH, follicle stimulating hormone; HT, hormone therapy; Kg, kilograms; M, meters; SHBG, steroid hormone binding globulin; SWAN, Study of Women’s Health Across the Nation; T, testosterone
